# Lyconadins G and H, Two Rare Lyconadin-Type *Lycopodium* Alkaloids from *Lycopodium complanatum*

**DOI:** 10.1007/s13659-016-0111-9

**Published:** 2016-10-25

**Authors:** Jin-Tang Cheng, Zhi-Jun Zhang, Xiao-Nian Li, Li-Yan Peng, Huai-Rong Luo, Xing-De Wu, Qin-Shi Zhao

**Affiliations:** 1 State Key Laboratory of Phytochemistry and Plant Resources in West China, Kunming Institute of Botany, Chinese Academy of Sciences, Kunming, 650201 People’s Republic of China; 2 Institute of Chinese Materia Medica, China Academy of Chinese Medical Sciences, Beijing, 100700 People’s Republic of China

**Keywords:** *Lycopodium* alkaloids, *Lycopodium complanatum*, Lyconadin

## Abstract

**Abstract:**

Two rare lyconadin-type *Lycopodium* alkaloids, lyconadins G (**1**) and H (**2**), together with four known ones (**3**–**6**), were isolated from *Lycopodium complanatum*. The structures were determined on the basis of their spectroscopic analyses, and the absolute configuration of **1** was established by an X-ray crystallographic analysis. It is the first time to establish the absolute configuration of lyconadin-type *Lycopodium* alkaloid by an X-ray diffraction experiment. In addition, these findings may provide more information for the biosynthesis of lyconadins.

**Graphical Abstract:**

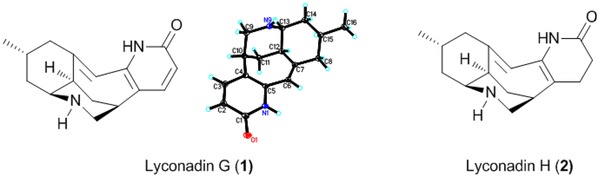

**Electronic supplementary material:**

The online version of this article (doi:10.1007/s13659-016-0111-9) contains supplementary material, which is available to authorized users.

## Introduction

**Fig. 1:**
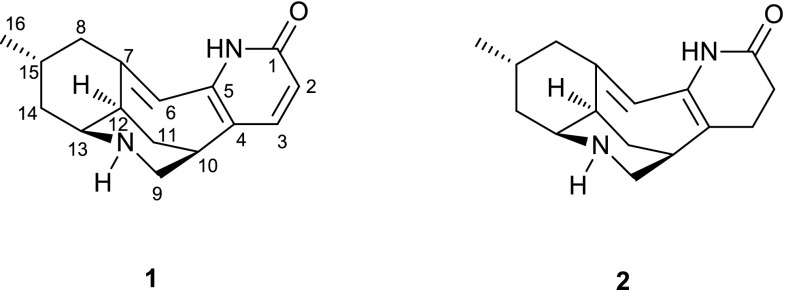
Structures of lyconadins G (**1**) and H (**2**)

The *Lycopodium* alkaloids are a unique family of complex natural products that have garnered long-standing interest from chemists as challenging targets for total synthesis [1–5], due to their fascinating structure complexity and wide-ranging biological activities [6–8]. Of these, huperzine A (Hup A), isolated from the Chinese folk medicinal herb *Qian Ceng Ta* [whole plant of *Huperzia serrata* (Thunb.ex Murray) Trev.], is a promising agent to treat Alzheimer’s disease with highly specific and potent inhibitory activity against acetylcholinesterase (AChE) [9]. As a consequence, these structurally complex *Lycopodium* alkaloids have inspired many research groups to study the chemical constituents in the *Lycopodium* plants [10–13]. *Lycopodium complanatum* (L.) Holub, mainly distributed in the temperate and subtropical regions of the world [14], was used as a traditional Chinese herbal medicine for the treatment of arthritic pain, quadriplegia, and contusion [15]. Previously, lycospidine A, a unique C_15_N *Lycopodium* alkaloid possibly derived from proline instead of the lysine biosynthetically, was isolated from this species [16]. As a part of an ongoing research program to discover more *Lycopodium* alkaloids with fascinating structures and bioactivities serving as lead compounds for drug discovery [17, 18], two new rare lyconadins, termed lyconadins G and H (**1** and **2**), were isolated from *L. complanatum* (Fig. 1), together with four known ones, lyconadin A (**3**) [19] and lyconadins C–E (**4**–**6**) [20, 21]. To the best of our knowledge, although more than 300 *Lycopodium* alkaloids have been reported so far [22–25], only six ones belong to the lyconadin-type [19–21, 26]. In this paper, we describe the isolation and structure elucidation of lyconadins G (**1**) and H (**2**).

## Results and Discussion

The air-dried and powdered whole plant of *L. complanatum* was extracted with MeOH three times. The extract was partitioned between EtOAc and 1 % HCl/H_2_O. The pH of the water-soluble portion was adjusted to pH 9 with saturated Na_2_CO_3_ aq. Then, it was extracted with CHCl_3_ to afford an alkaloidal extract. Further column chromatography over MCI gel, silica gel, and semipreparative HPLC led to the isolation of compounds **1** (7 mg), **2** (2 mg), **3** (11 mg), **4** (8 mg), **5** (4 mg) and **6** (6 mg).

**Fig. 2:**
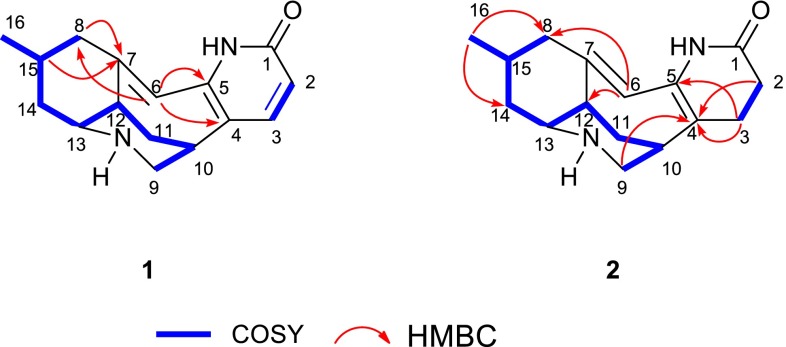
Key ^1^H-^1^H COSY and HMBC correlations of lyconadins G (**1**) and H (**2**)

Lyconadin G (**1**), obtained as colorless blocks, was assigned to have a molecular formula of C_16_H_20_N_2_O by HR-EI-MS (*m/z* 256.1576, calcd 256.1576) and ^13^C NMR spectroscopic data (Table 1). The IR absorption implied the presence of amide carbonyl (1647 cm^−1^) functionality. The ^13^C NMR and DEPT spectra (Table 1) exhibited 16 carbon resonances, including four quaternary carbons (one carbonyl and three olefinic), seven methines [including three olefinic (*δ*_C_ 120.7, 116.8, and 146.6) with corresponding protons as singlet signal at *δ*_H_ 6.28 and two mutually coupled signals at *δ*_H_ 6.34 and 7.30, respectively, in the ^1^H NMR spectrum], four methylenes, and one methyl. Based on the above evidence, the structure of **1** was determined to be similar to that of lyconadin C (**4**) [20]. Comparison of the NMR data of **1** and **4** suggested that the most obvious differences were the loss of two *sp* ^3^ carbons (one *sp* ^3^ methine carbon and one *sp* ^3^ methylene carbon) and the presence of one more trisubstituted double bond in **1**. The ^1^H-^1^H COSY spectrum of **1** (Fig. 2) showed two partial structures a (C-2/C-3), b (C-9 to C-16 and C-8/C-15), implying that the additional double bond in **1** was located between C-6 and C-7. The HMBC correlations from H-15 and H_2_-8 to C-7 and from H-6 to C-4, C-5, and C-8 (Fig. 2) further supported the planar structure of **1** as shown in Fig. 2.

**Table 1:** ^1^H (600 MHz) and ^13^C (150 MHz) NMR data of **1** and **2** in CD_3_OD (*δ* in ppm, *J* in Hz)

	**1**		**2**	
	*δ*_H_ (mult, *J* in Hz)	*δ*_C_	*δ*_H_ (mult, *J* in Hz)	*δ*_C_
1		164.9 s		173.7 s
2	6.34 (d, 9.1)	116.8 d	2.42 (2H, m)	30.7 t
3	7.30 (d, 9.1)	146.6 d	2.49 (2H, m)	28.7 t
4		124.8 s		120.2 s
5		142.3 s		131.3 s
6	6.28 (s)	120.7 d	5.85 (s)	123.1 d
7		154.6 s		145.5 s
8a	2.39 (dd, 11.5, 2.9)	49.2 t	2.37 (dd, 11.8, 2.8)	48.2 t
8b	1.96 (t, 11.5)		2.00 (t, 11.8)	
9a	3.22 (2H, overlapped)	55.4 t	3.53 (d, 13.0)	52.0 t
9b			3.44 (dd, 13.0, 4.9)	
10	2.77 (m)	36.9 d	2.69 (m)	35.8 d
11a	2.20 (m)	29.3 t	2.24 (m)	28.5 t
11b	2.07 (m)		2.21 (m)	
12	2.69 (m)	43.2 d	2.85 (m)	40.9 d
13	3.22 (overlapped)	60.7 d	3.66 (m)	61.8 d
14a	1.81 (m)	40.7 t	1.98 (m)	38.1 t
14b	1.52 (td, 13.4, 4.3)		1.73 (ddd, 15.0, 13.0, 4.3)	
15	1.67 (m)	35.2 d	1.85 (m)	34.2 d
16	0.99 (d, 6.3)	22.4 q	1.05 (d, 6.4)	22.0 q

^1^H- and ^13^C-NMR were recorded in CDCl_3_ and CD_3_OD

**Fig. 3:**
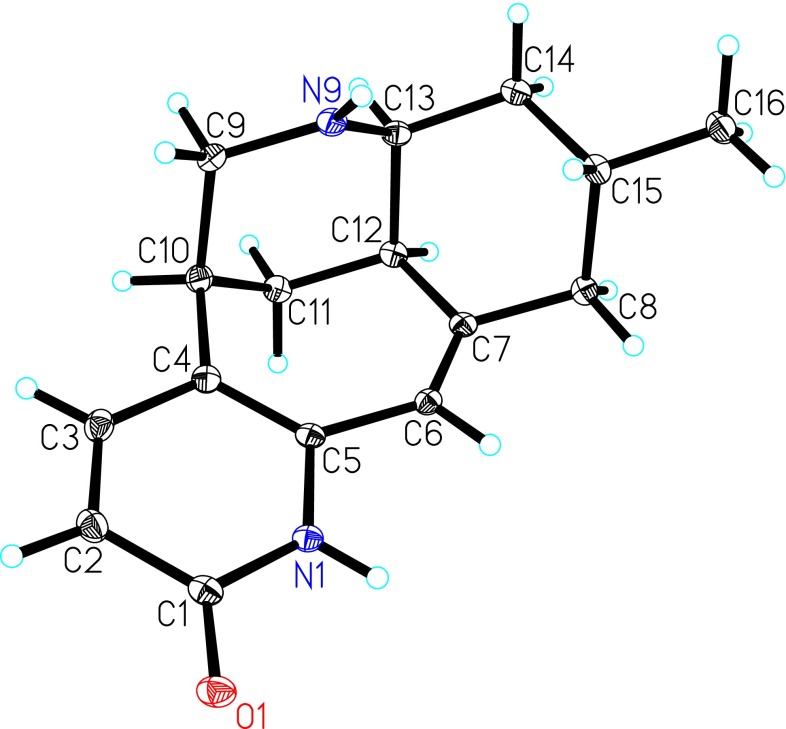
The X-ray crystal structure of lyconadin G (**1**)

In the ROESY spectrum of **1**, the ROESY correlations of H-12/H-8b, H-12/H-14b suggested that they were in the same orientations. The fact that C-16 was in an equatorial position was deduced from the ROESY cross-peak of H-8b/H_3_-16. However, there were no solid proof which can be used to establish the relative configurations of C-10 and C-13. Fortunately, crystals suitable for single-crystal X-ray diffraction of **1** was obtained from CHCl_3_–MeOH. The structure of **1** was confirmed by single-crystal X-ray diffraction using the anomalous scattering of CuK radiation with a Flack parameter of 0.2(2) (Fig. 3). Its absolute configuration was unambiguously assigned as 10*S*, 12*R*, 13*S*, 15*R*.

Lyconadin H (**2**), showed the pseudomolecular ion peak at *m/z* 259 [M + H]^+^ in the ESI–MS, and the molecular formula, C_16_H_22_N_2_O, was established by HR-EI-MS [*m/z* 258.1732 Calc. 258.1732]. The IR absorption implied the presence of amide carbonyl (1680 cm^−1^) functionality. The ^13^C NMR (Table 1) spectrum of **2** gave signals due to four quaternary carbons (one carbonyl, three olefinic), five methines (including one olefinic (*δ*_C_ 123.1) with corresponding proton as a singlet signal at *δ*_H_ 5.85 in the ^1^H NMR spectrum), six methylenes, and one methyl, implying that the structure of **2** was similar to that of lyconadin G (**1**). The sole difference was the loss of a 1,2-disubstituted double bond between C-2 and C-3, which was confirmed by the HMBC correlations of H_2_-2 and H_2_-3 with C-4, and of H_2_-3 with C-5, as well as the ^1^H-^1^H COSY correlation between H_2_-2 and H_2_-3.

**Fig. 4:**
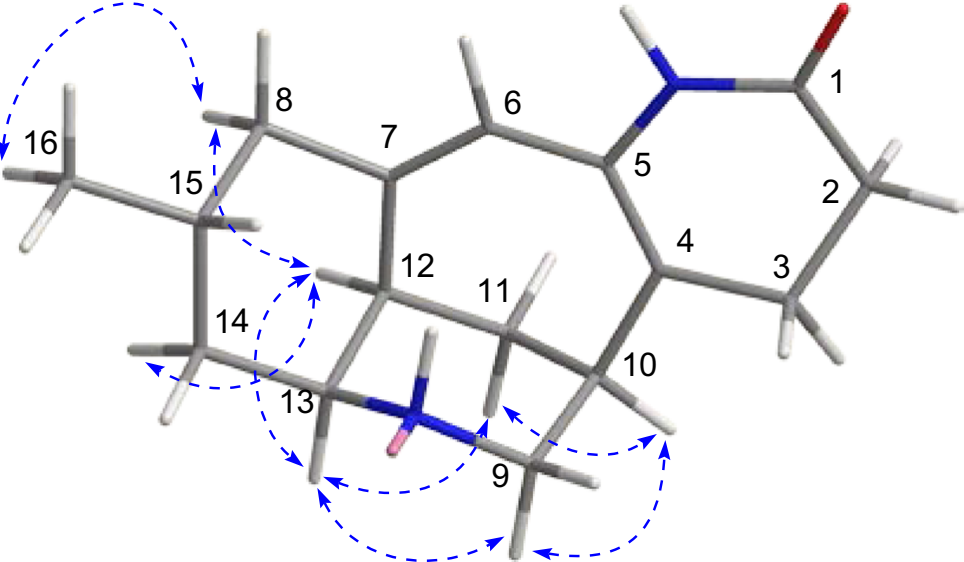
Key ROESY correlations of lyconadin H (**2**)

The relative configuration of **2** was deduced from ROESY correlations as shown in computer-generated 3D drawing (Fig. 4). ROESY correlations of H-12/H-8b, H-12/H-14b and H-8b/H_3_-16 revealed they took the same orientations. A *cis*-fused ring junction between the cyclohexane ring and a piperidine ring (C-9–C-13 and N-9) was inferred from the coupling constant of ^3^ *J*_H-13/H-14b_ value (4.3 Hz) and ROESY correlation of H-12/H-13 [19, 20]. ROESY cross-peaks of H-13/H-11a and H-13/H-9a indicated the chair form of the piperdine ring (C-9–C-13 and N-9). The relative configuration of H-10 was elucidated to be equatorial by the ROESY correlations of H-10/H-9a, and H-10/H-11a. Moreover, the specific rotation between **2** (+95.6) and **1** (+126.6) indicated they have the same absolute configuration. Accordingly, the structure of **2** was determined as shown in Fig. 4.

**Scheme 1:**
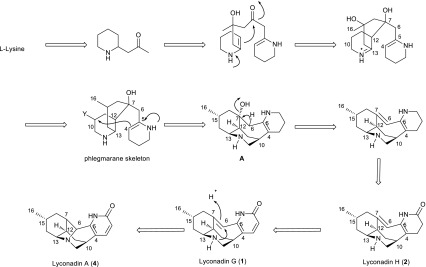
Plausible biogenetic pathway for compounds **1** and **2**

Among *Lycopodium* alkaloids, lyconadins are a unique family which contain intriguing tetra- or pentacyclic ring systems fused by the unique C-4–C-10 linkage and C-6–N-9 bond, and thus offer a new horizon for organic chemists [27–29]. Biogenetically, the discovery of two new compounds (**1** and **2**), together with four known lyconadins (**3**–**6**) from the title plant can provide some new insight into the biosynthesis of lyconadins [19, 20]. Possible biogenetic pathway for lyconadins G (**1**), H (**2**), and A (**3**) is proposed in Scheme 1. As illustrated in Scheme 1, the key intermediate A might arise from phlegmarane skeleton by C-4 and C-10 carbon bond formation and subsequent loss of H_2_O and oxidation to yield compounds **1** and **2**. In addition, the double bond located between C-6 and C-7 in **1** could serve as a suitable electrophile in the process of the desired C-6–N-9 bond formation to produce lyconadin A (**3**). The potent biological activities of **1** and **2** against AChE using the improved Ellman method [30, 31] were also evaluated. However, neither of them showed obvious activity (IC_50_ > 100 µM).

## Experimental

### General

Optical rotations were measured with a JASCO P-1020 polarimeter. UV spectra were recorded with a Shimadzu UV-2401A spectrophotometer. IR spectra were recorded on Bruker Tensor 27 spectrometer with a KBr disk. 1D and 2D NMR spectra were recorded on a Bruker Avance III 600 spectrometer. Chemical shifts were reported using TMS as the internal standard. ESI–MS were recorded on Shimadzu UPLC-IT-TOF-MS instrument and HREIMS spectra were measured with a Waters AutoSpec Premier P776 mass spectrometer. Column chromatography (CC) was performed on silica gel (90–200 µm; Qingdao Haiyang Chemical Co. Ltd., Qingdao, China), and MPLC was performed on a Lisui EZ Purify III System packed with RP-18 silica gel (40–63 μm, Merck, 71 Darmstadt, Germany) columns. Precoated silica gel GF254 plates (Qingdao Haiyang Chemical Co. Ltd.) were used for thin-layer chromatography (TLC). Preparative and semipreparative HPLC was performed on Shimadzu LC-8A equipped with a Shimadzu PRC-ODS (K) column and Agilent 1100 apparatus equipped with a Zorbax SB-C-18 75 (Agilent, 9.4 mm × 25 cm) column, respectively. Fractions were monitored by TLC and spots were visualized by Dragendorff’s reagent.

### Plant Materials

The club moss *L. complanatum* was collected from Jinping County, Yunnan Province, People’s Republic of China in April, 2011. The plant was identified by Prof. Xiao Cheng (Kunming Institute of Botany, Chinese Academy of Sciences). And its voucher specimen (201104M) was deposited at the State Key Laboratory of Phytochemistry and Plant Resources in West China, Kunming Institute of Botany, CAS.

### Extraction and Isolation

The club moss *L. complanatum* (L.) Holub (100 kg) was chopped into sections and extracted with methanol under reflux for three times (4, 3, 3 h, respectively,). The resultant extract was partitioned between EtOAc and 1 % HCl/H_2_O solution to afford ethyl acetate and water soluble fractions, respectively. The water-soluble fractions was adjusted to pH 9 by saturated Na_2_CO_3_, and then extracted with chloroform to give an alkaloidal extract (128 g). The alkaloidal extract was subjected to a MCI gel CC (eluted with 20 % MeOH to 100 % MeOH) to afford fractions I-III. Fraction I (43.7 g) was further chromatographed over repeated silica gel columns (CHCl_3_/MeOH) to give subfractions Ia-Ic. Subfractions Ia was subjected to CC (SiO_2_, CHCl_3_/MeOH, gradient system) and finally purified by a C_18_ HPLC (MeOH/H_2_O/TFA, 20:80:0.1) to afford compounds **1** (7 mg) and **2** (2 mg). From subfraction Ic, compounds **3** (11 mg) and **4** (8 mg) were obtained after purified by repeated silica gel columns (CHCl_3_/MeOH) and semipreparative HPLC (MeOH/H_2_O/TFA, 18:82:0.1). Fraction II (35.0 g) was subjected to CC (SiO_2_, CHCl_3_/MeOH, gradient system) and repeated crystallization to give **5** (4 mg). Compound **6** (6 mg) was obtained after repeated chromatography and semipreparative HPLC (MeOH/H_2_O/TFA, 22:78:0.1) from fraction II.

#### Lyconadin G (**1**)

Colorless blocks; ${[{\rm \alpha}]}^{23.3}_{\rm D}$ + 126.6 (*c* = 0.2, MeOH); UV (MeOH) *λ*_max_ (log *ε*) 206 (4.20), 252 (3.75) 345 (3.94) nm; IR (neat) *ν*_max_ 3432, 2949, 2924, 2868, 1647, 1595, 1454, 1120 cm^−1^; for ^1^H, ^13^C NMR data see Table 1; HREIMS *m/z* 256.1576 (calcd for C_16_H_20_N_2_O, 256.1576).

#### Crystal Data for Lyconadin G (**1**)

C_16_H_22_N_2_O_2_ (C_16_H_20_N_2_O.H_2_O), MW = 274.36; orthorhombic, space group *P*2_1_2_1_2_1_; *a* = 8.2185(2) Å, *b* = 9.3774(2) Å, *c* = 18.2826(3) Å, *α* = *β* = *γ* = 90°, V = 1409.01(5) Å^3^, *Z* = 4, *d* = 1.293 g/cm^3^, crystal dimensions 0.20 × 0.25 × 0.75 mm were used for measurement on a Bruker APEX DUO with a graphite monochromator CuK_α_ radiation. The total number of reflections measured was 6967, of which 2513 were observed, |F|^2^ ≥ 2σ|F|^2^. Final indices: R_1_ = 0.0337, wR_2_ = 0.0898 (w = 1/σ|F|^2^). Flack parameter = 0.2(2). The structure of **1** was solved by direct method SHELXS-97. Crystallographic data center (deposition number: CCDC 1507256).

#### Lyconadin H (**2**)

Colorless oil; [α]_D_^23.3^  + 95.6 (*c* = 0.1, MeOH); UV (MeOH) *λ*_max_ (log *ε*) 223 (3.76), 291 (3.19) nm; IR (neat) *ν*_max_ 3433, 2928, 1680, 1437, 1207, 1135, 801, 723 cm^−1^; for ^1^H, ^13^C NMR data see Table 1; HREIMS *m/z* 258.1732 (calcd for C_16_H_22_N_2_O, 258.1732).

### Acetylcholinesterase Inhibitory Activity

AChE inhibitory activity of lyconadins G (**1**) and H (**2**) was assayed by the spectrophotometric method developed by Ellman with slightly modification. *S*-Acetylthiocholine iodide, *S*-butyrylthiocholine iodide, 5,5′-dithio-bis-(2-nitrobenzoic) acid (DTNB, Ellman’s reagent), AChE derived from human erythrocytes were purchased from Sigma Chemical. Compounds were dissolved in DMSO. The reaction mixture (totally 200 μL) containing phosphate buffer (pH 8.0), test compound (50 μM), and AChE (0.02 U/mL), was incubated for 20 min (30 °C). Then, the reaction was initiated by the addition of 40 μL of solution containing DTNB (0.625 mM) and acetylthiocholine iodide (0.625 mM) for AChE inhibitory activity assay, respectively. The hydrolysis of acetylthiocholine was monitored at 405 nm every 3 min for 1 h. Tacrine was used as positive control with final concentration of 0.333 μM. All the reactions were performed in triplicate. The percentage inhibition was calculated as follows: % inhibition = (E − S)/E × 100 (E is the activity of the enzyme without test compound and S is the activity of enzyme with test compound).

## Electronic supplementary material

Below is the link to the electronic supplementary material.

Supplementary material 1 (DOCX 5689 kb)
