# Supplementary material for: Lyconadins G and H, Two Rare Lyconadin-Type Lycopodium Alkaloids from Lycopodium complanatum
Source: Nat Prod Bioprospect. 2016 Oct 25;6(6):279–84. doi: 10.1007/s13659-016-0111-9 (PMC5136372; doi:10.1007/s13659-016-0111-9)

Lyconadins G and H, Two Rare Lyconadin-type *Lycopodium* Alkaloids from *Lycopodium complanatum*

Jin-Tang Cheng Zhi-Jun Zhang Xiao-Nian Li Li-Yan Peng Huai-Rong Luo Xing-De Wu Qin-Shi Zhao

J.-T. Cheng Z.-J. Zhang X.-N. Li L-Y. Peng H.-R. Luo X.-D. Wu (🖂) Q-S. Zhao (🖂)

State Key Laboratory of Phytochemistry and Plant Resources in West China, Kunming Institute of Botany, Chinese Academy of Sciences, Kunming 650201, People’s Republic of China.

email: wuxingde@mail.kib.ac.cn, qinshizhao@mail.kib.ac.cn

J.-T. Cheng

Institute of Chinese Materia Medica, China Academy of Chinese Medical Sciences, Beijing 100700, People’s Republic of China

**Table of Contents**

[Figure S1. ^1^H NMR spectrum of lyconadin G 2](#_Toc456086027)

[Figure S2. DEPT spectrum of lyconadin G 3](#_Toc456086028)

[Figure S3. HREIMS spectrum of lyconadin G 4](#_Toc456086029)

[Figure S4. ^1^H-^1^H COSY NMR spectrum of lyconadin G 5](#_Toc456086030)

[Figure S5. HSQC NMR spectrum of lyconadin G 6](#_Toc456086031)

[Figure S6. HMBC NMR spectrum of lyconadin G 7](#_Toc456086032)

[Figure S7. ROESY NMR spectrum of lyconadin G 8](#_Toc456086033)

[Figure S8. ^1^H NMR spectrum of lyconadin H 9](#_Toc456086034)

[Figure S9. DEPT spectrum of lyconadin H 10](#_Toc456086035)

[Figure S10. HREIMS spectrum of lyconadin H 11](#_Toc456086036)

[Figure S11. ^1^H-^1^H COSY NMR spectrum of lyconadin H 12](#_Toc456086037)

[Figure S12. HSQC NMR spectrum of lyconadin H 13](#_Toc456086038)

[Figure S13. HMBC NMR spectrum of lyconadin H 14](#_Toc456086039)

[Figure S14. ROESY NMR spectrum of lyconadin H 15](#_Toc456086040)

# Figure S1. ^1^H NMR spectrum of lyconadin G


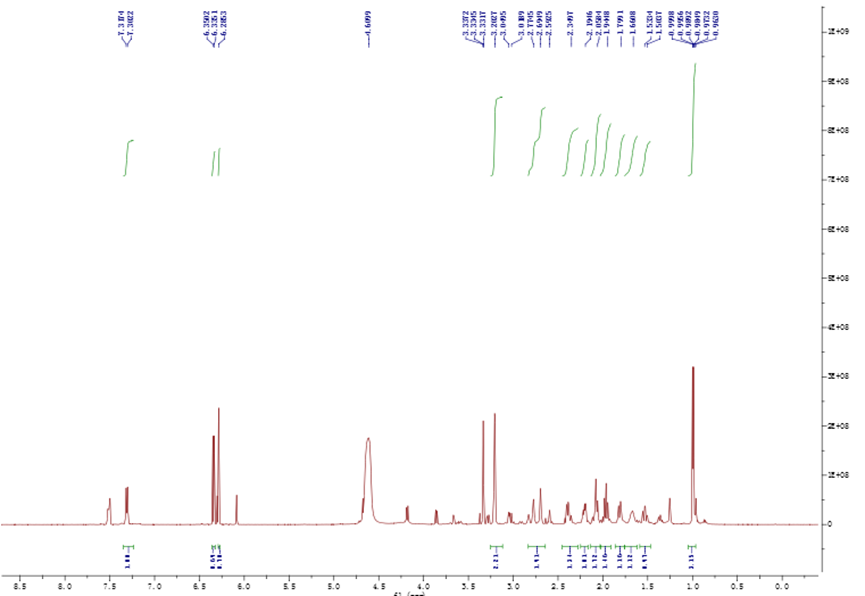


# Figure S2. DEPT spectrum of lyconadin G


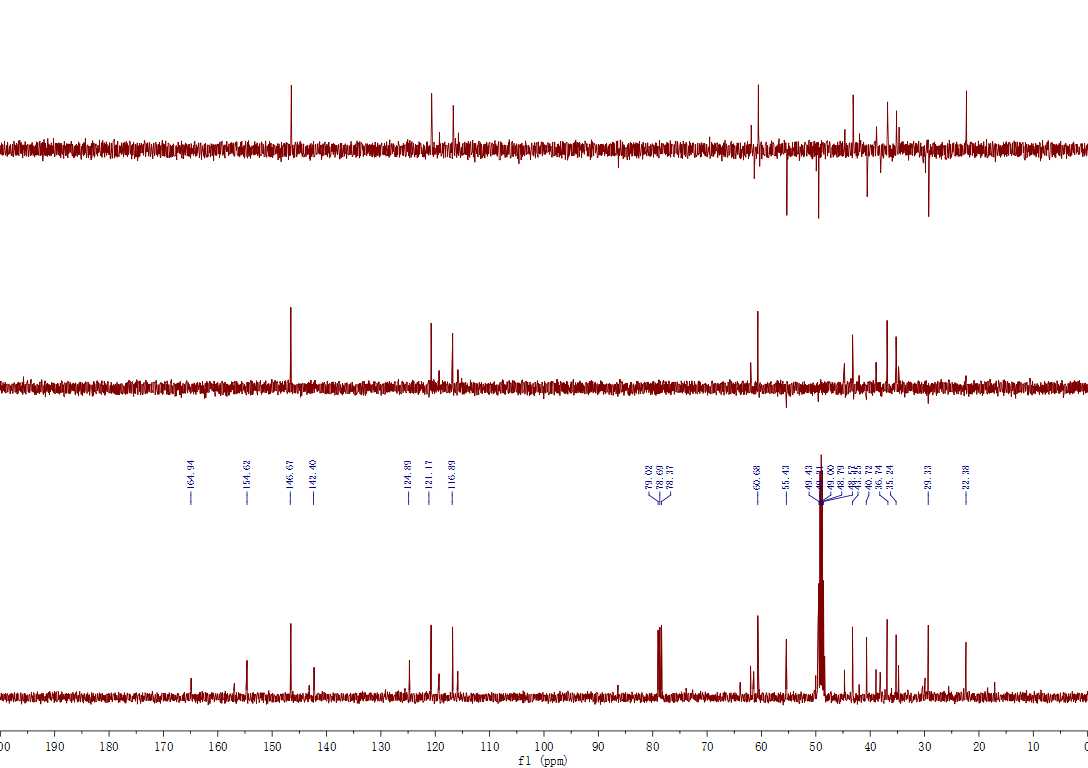


# Figure S3. HREIMS spectrum of lyconadin G
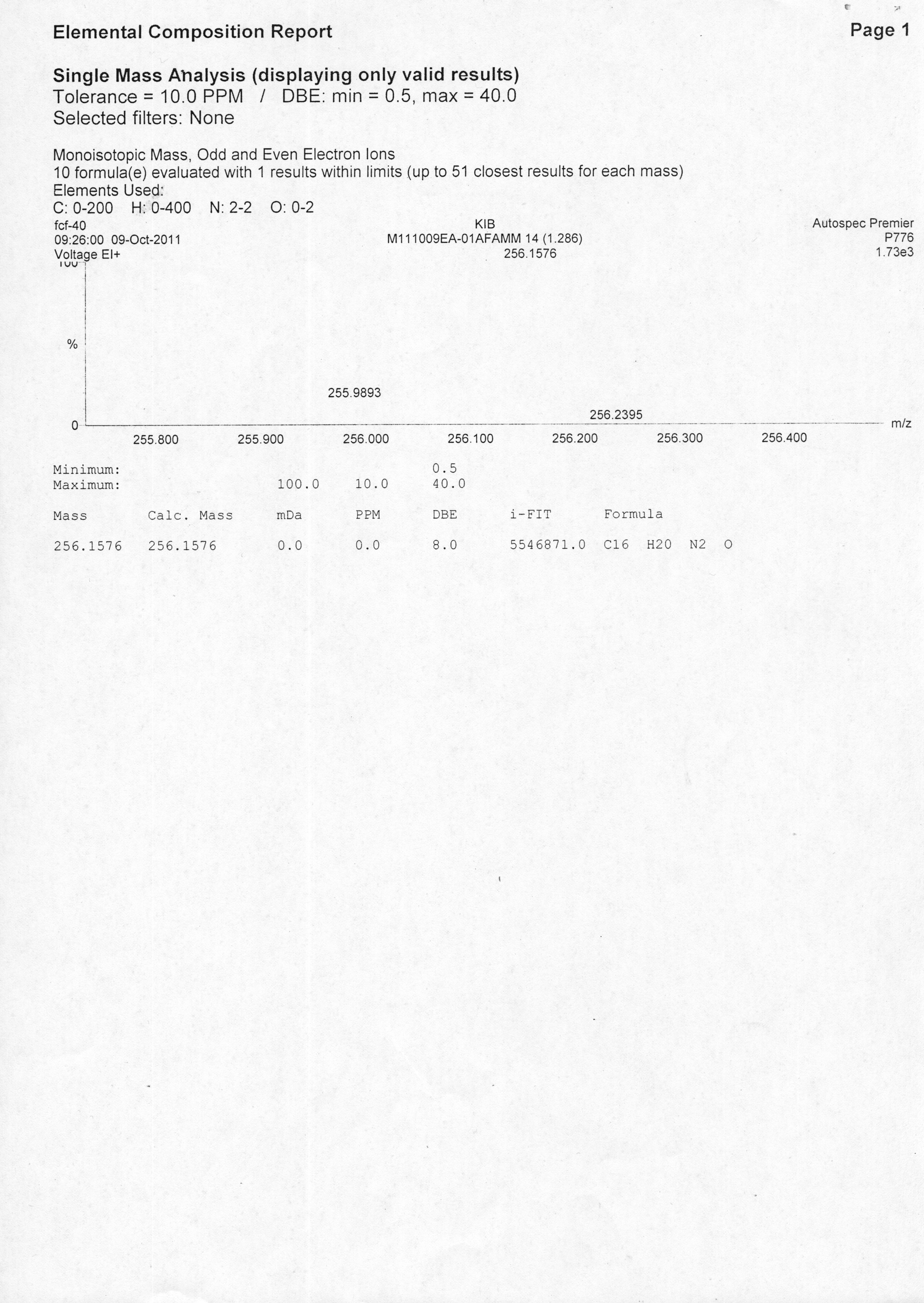


# Figure S4. ^1^H-^1^H COSY NMR spectrum of lyconadin G


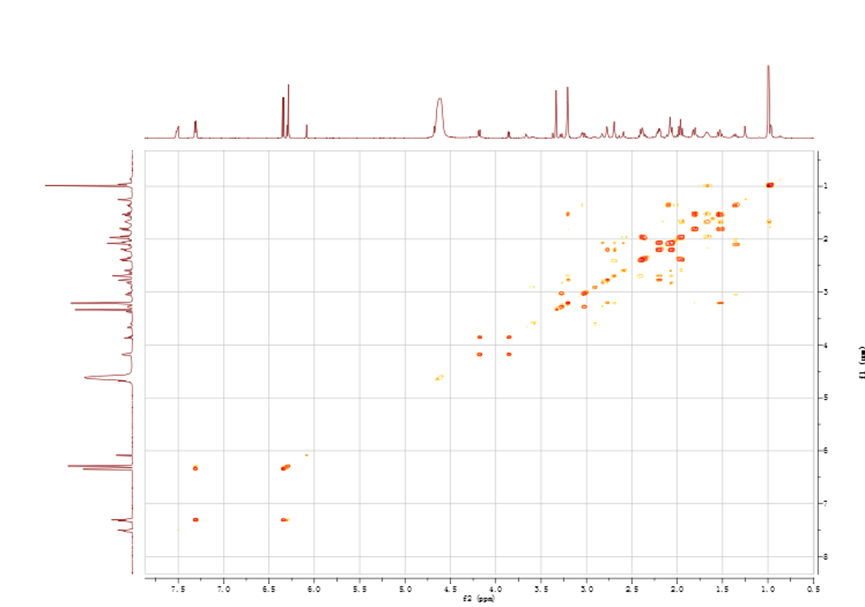


# Figure S5. HSQC NMR spectrum of lyconadin G


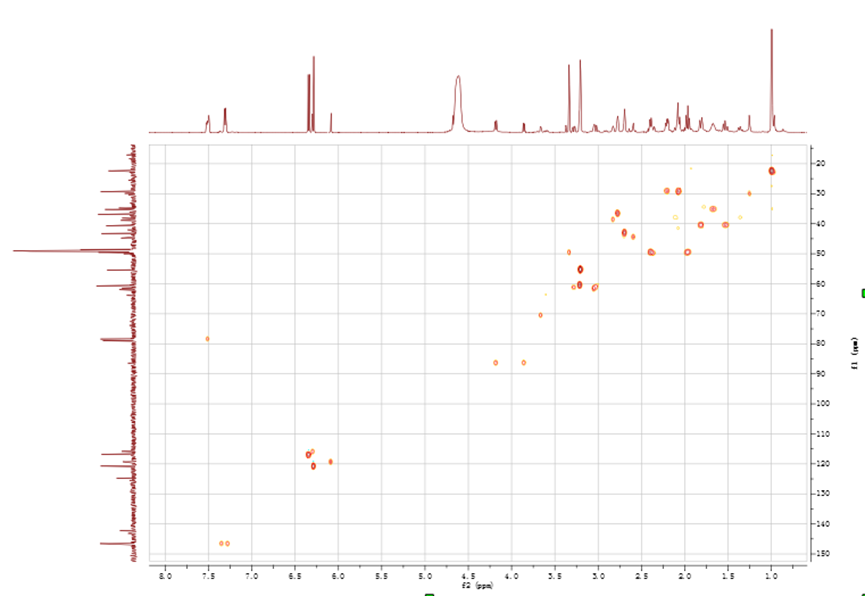


# Figure S6. HMBC NMR spectrum of lyconadin G


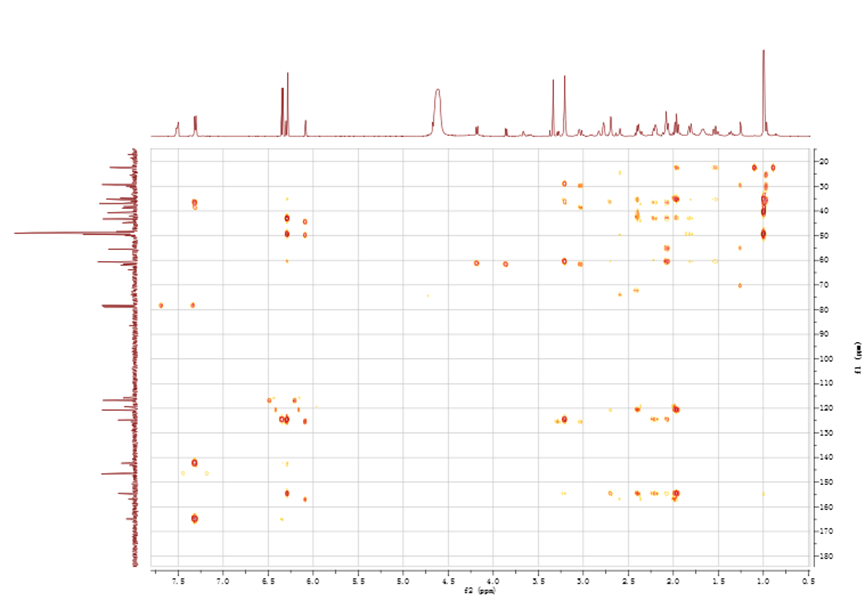


# Figure S7. ROESY NMR spectrum of lyconadin G


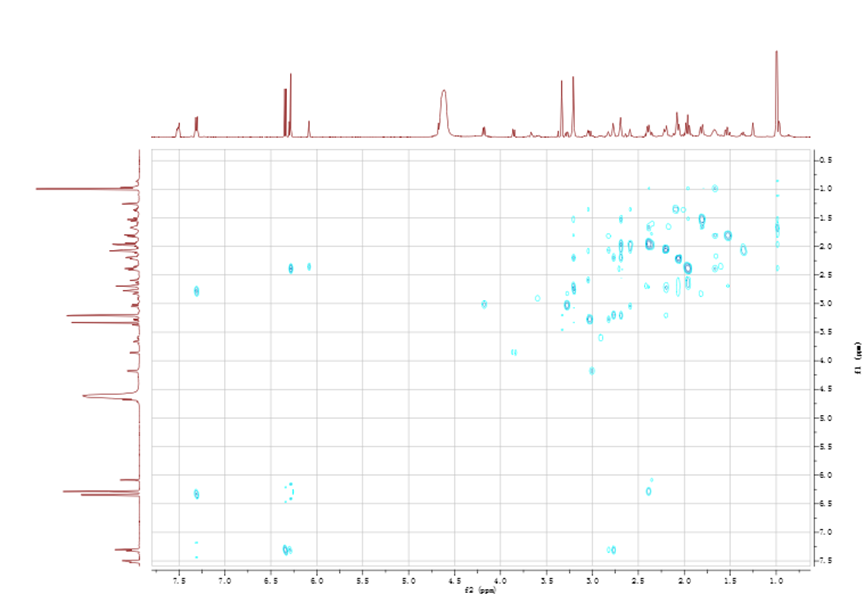


# Figure S8. ^1^H NMR spectrum of lyconadin H


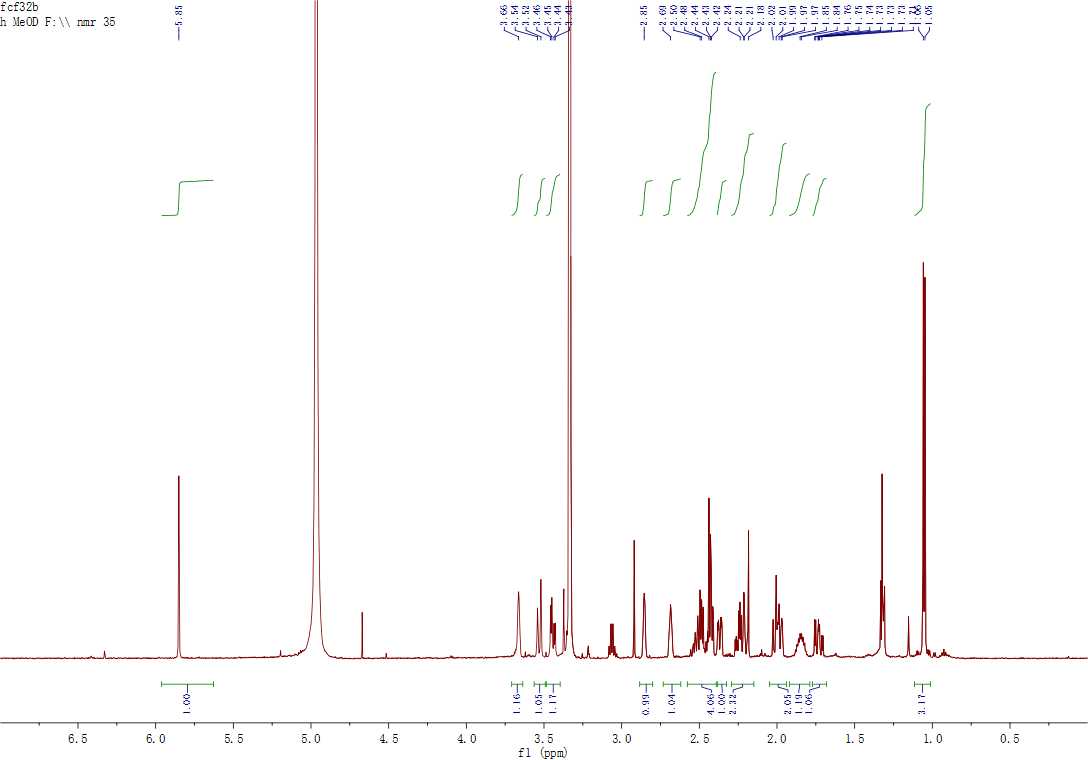


# Figure S9. DEPT spectrum of lyconadin H


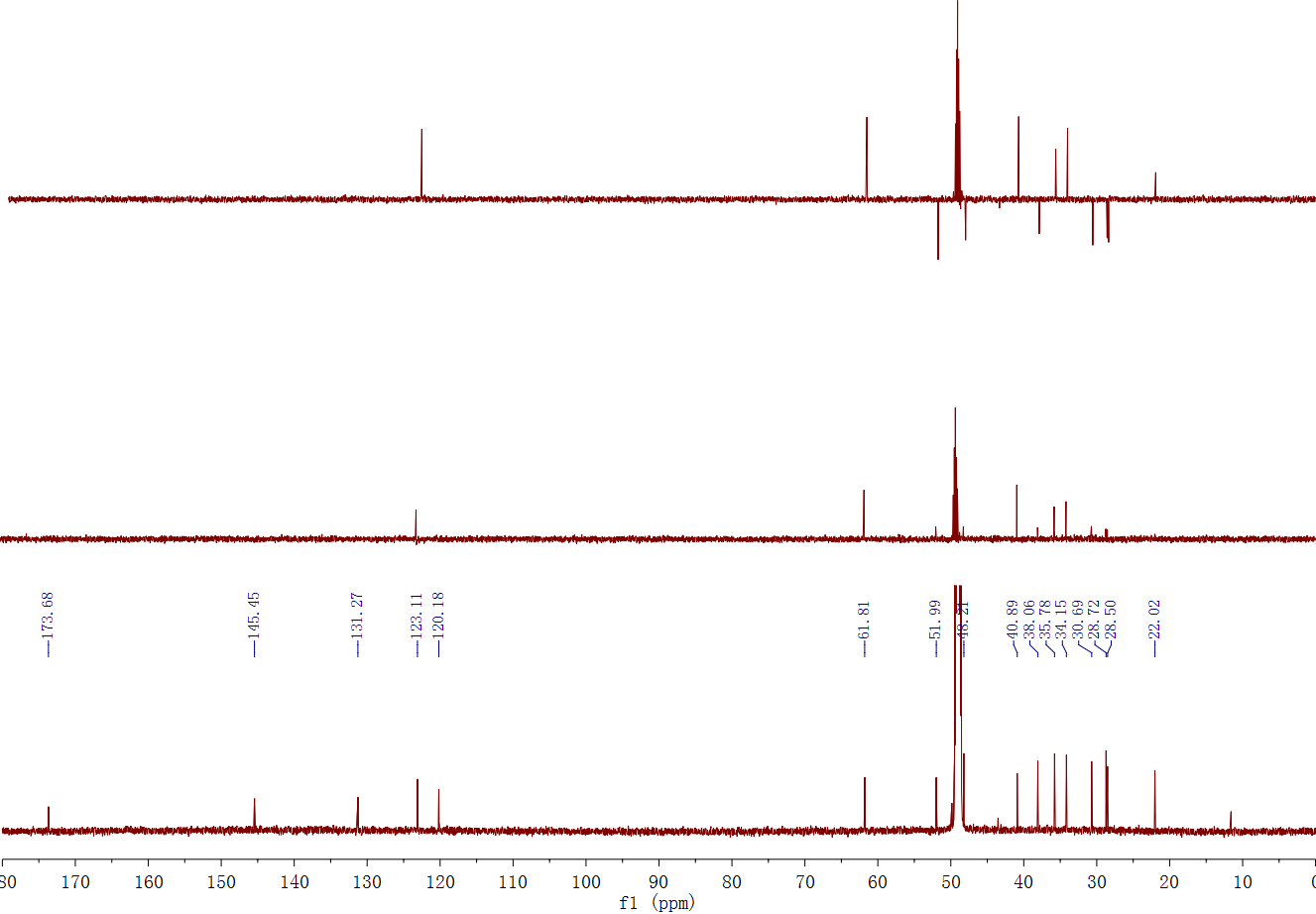


# Figure S10. HREIMS spectrum of lyconadin H


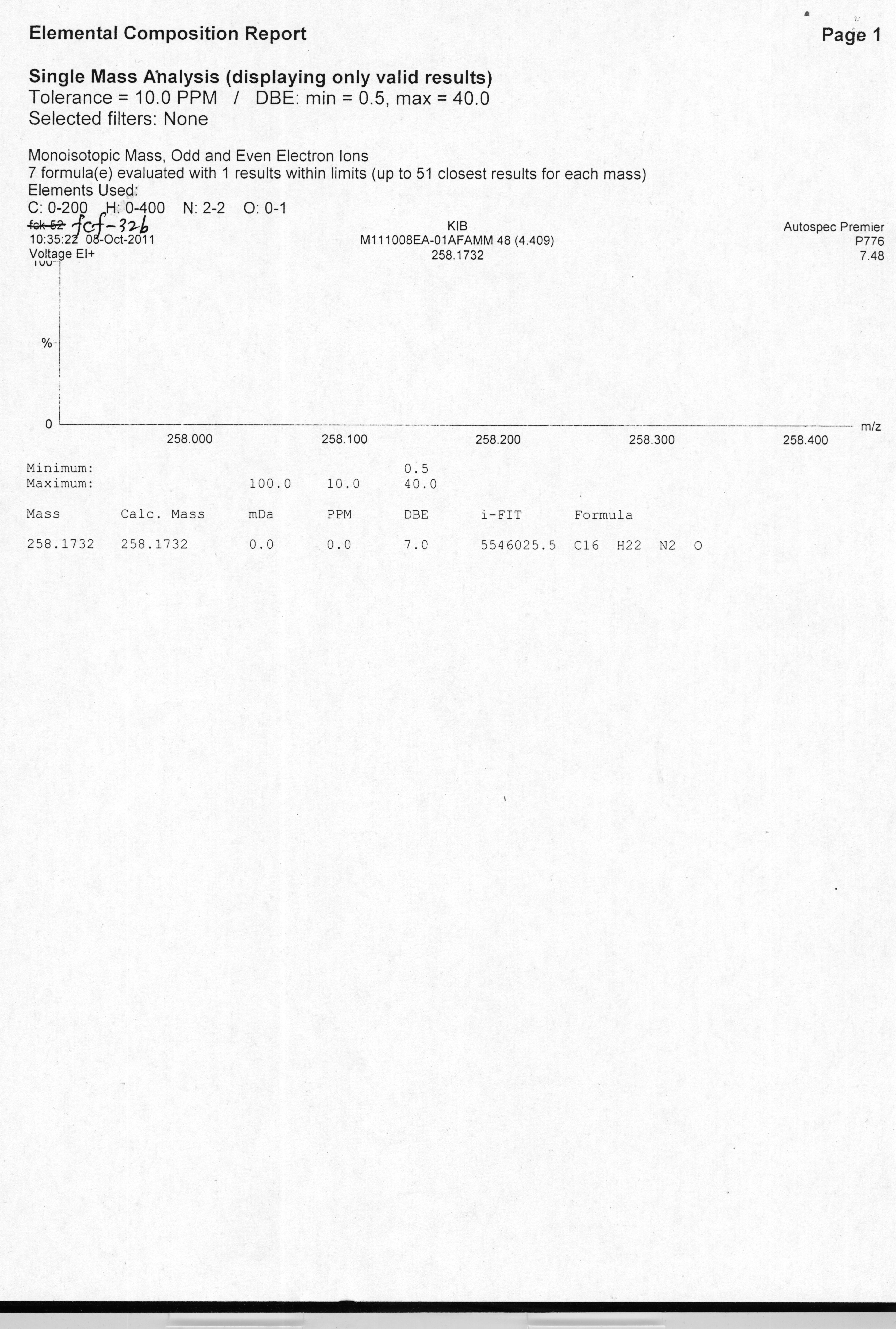


# Figure S11. ^1^H-^1^H COSY NMR spectrum of lyconadin H


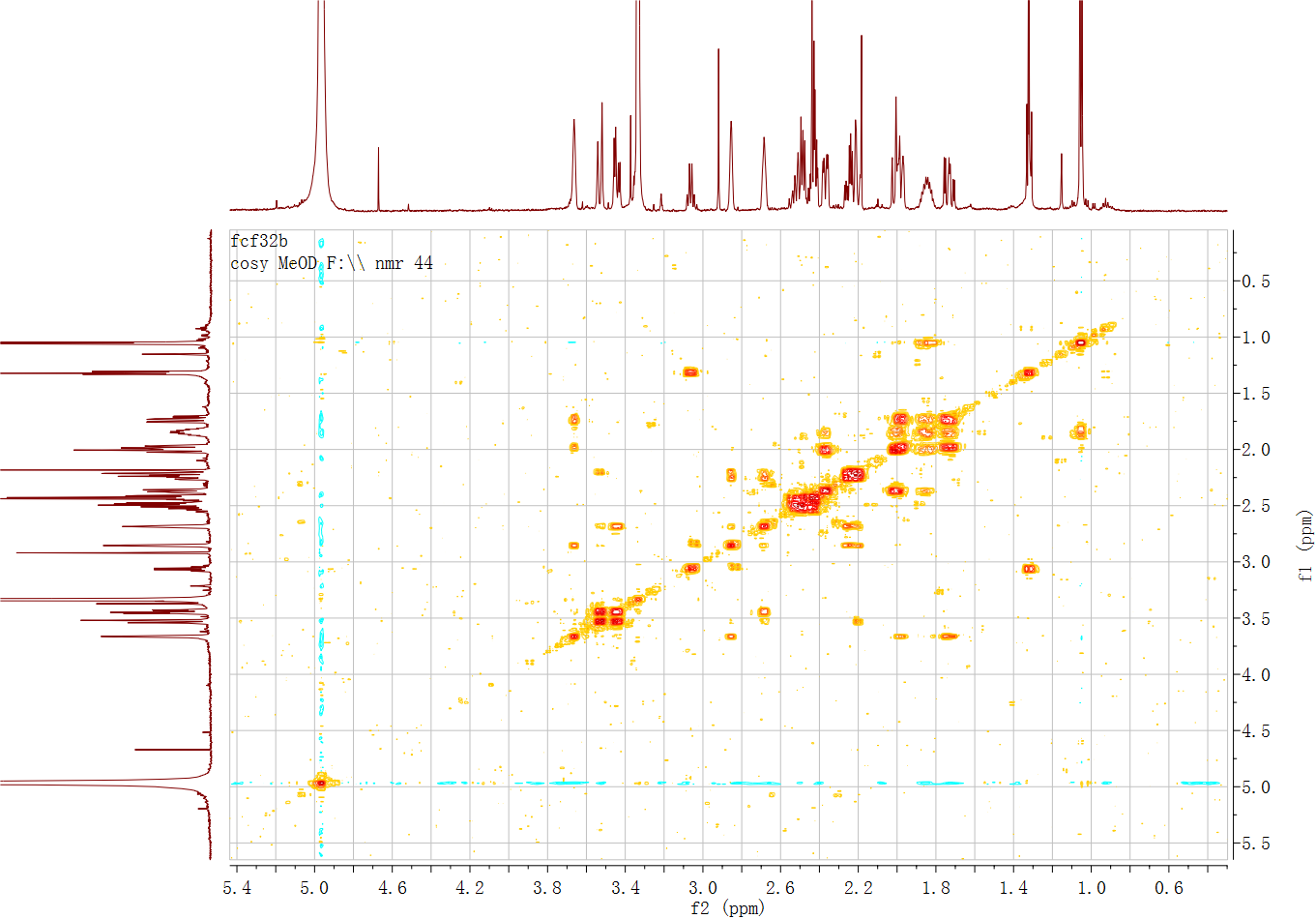


# Figure S12. HSQC NMR spectrum of lyconadin H


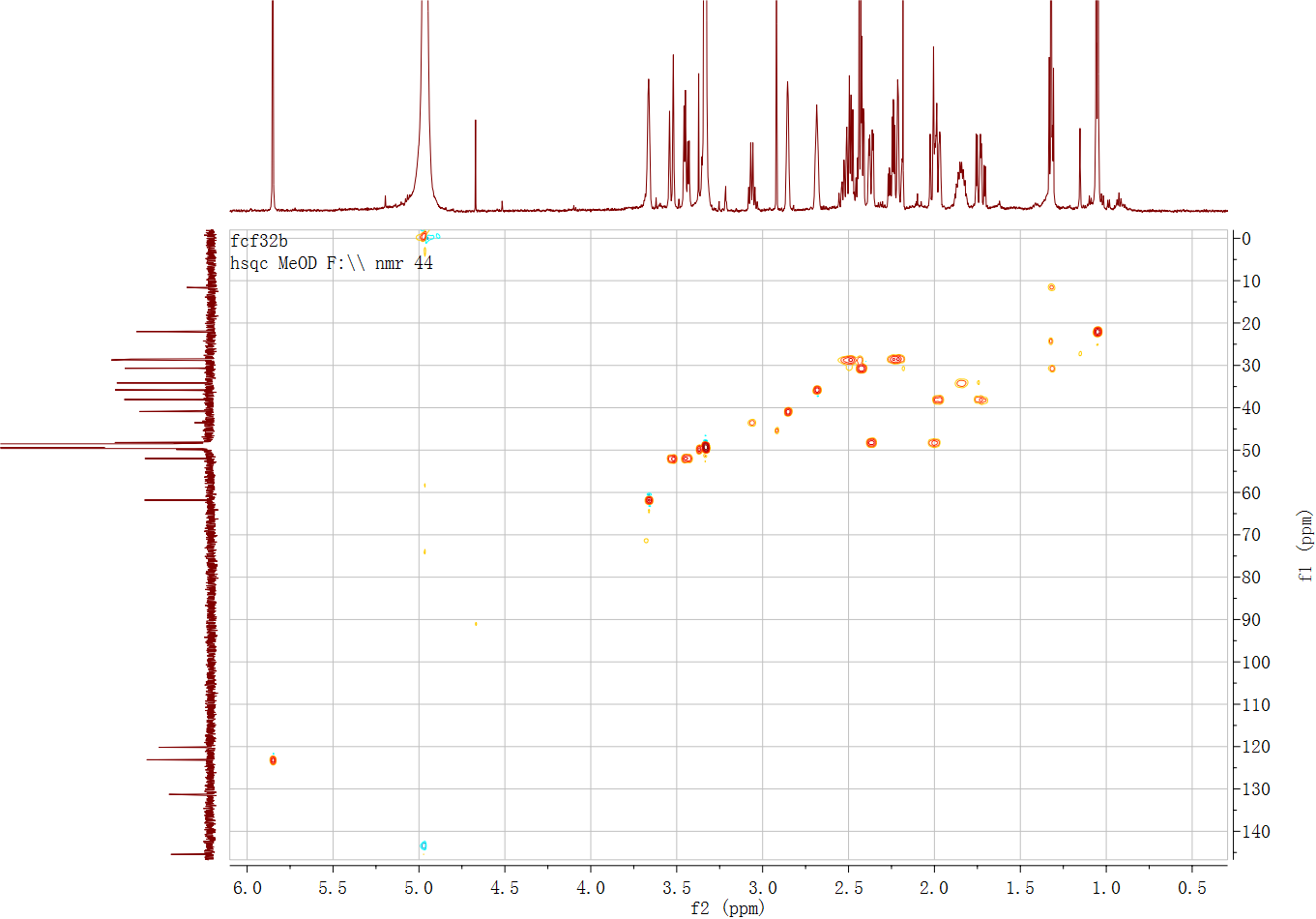


# Figure S13. HMBC NMR spectrum of lyconadin H


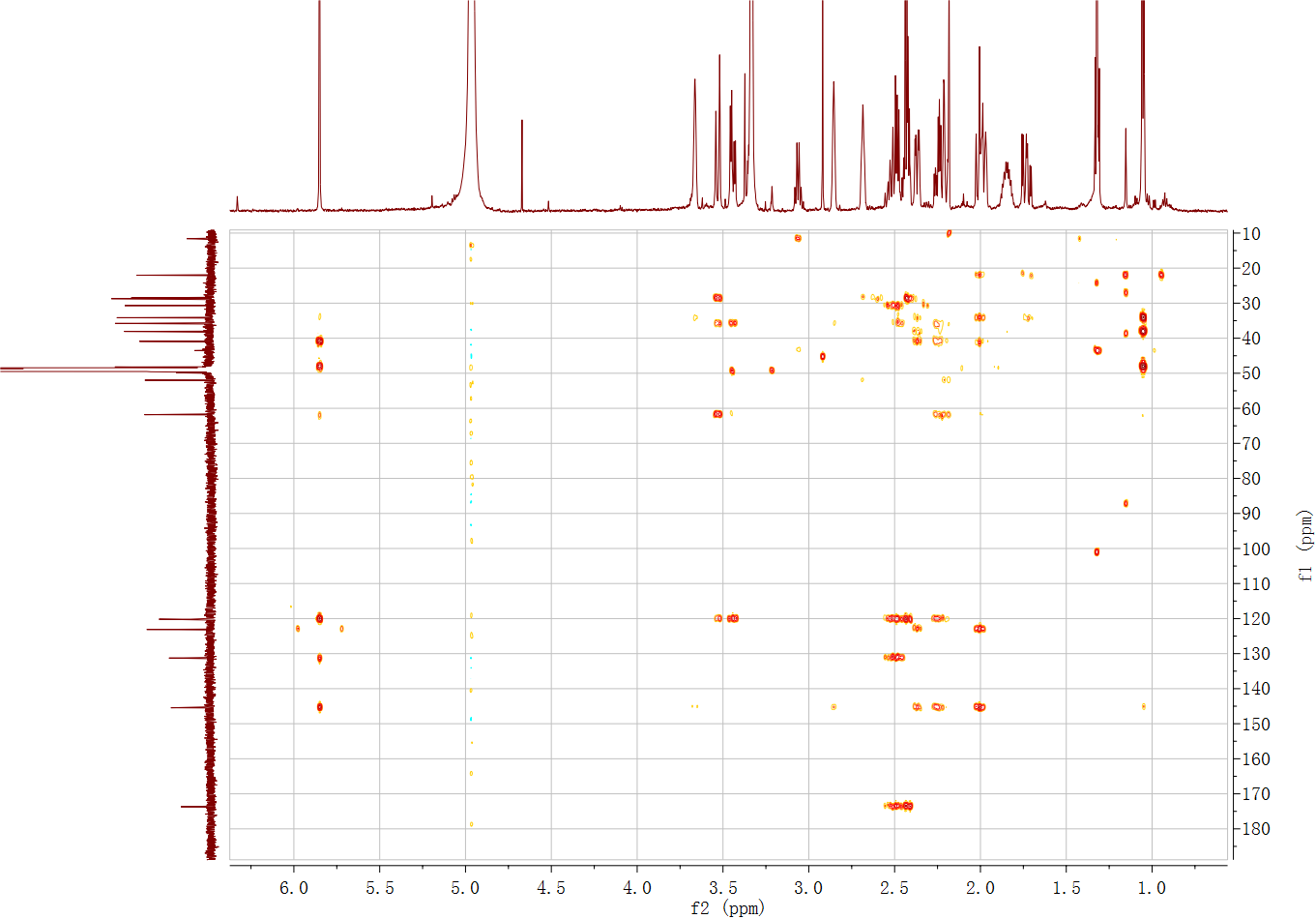


# Figure S14. ROESY NMR spectrum of lyconadin H


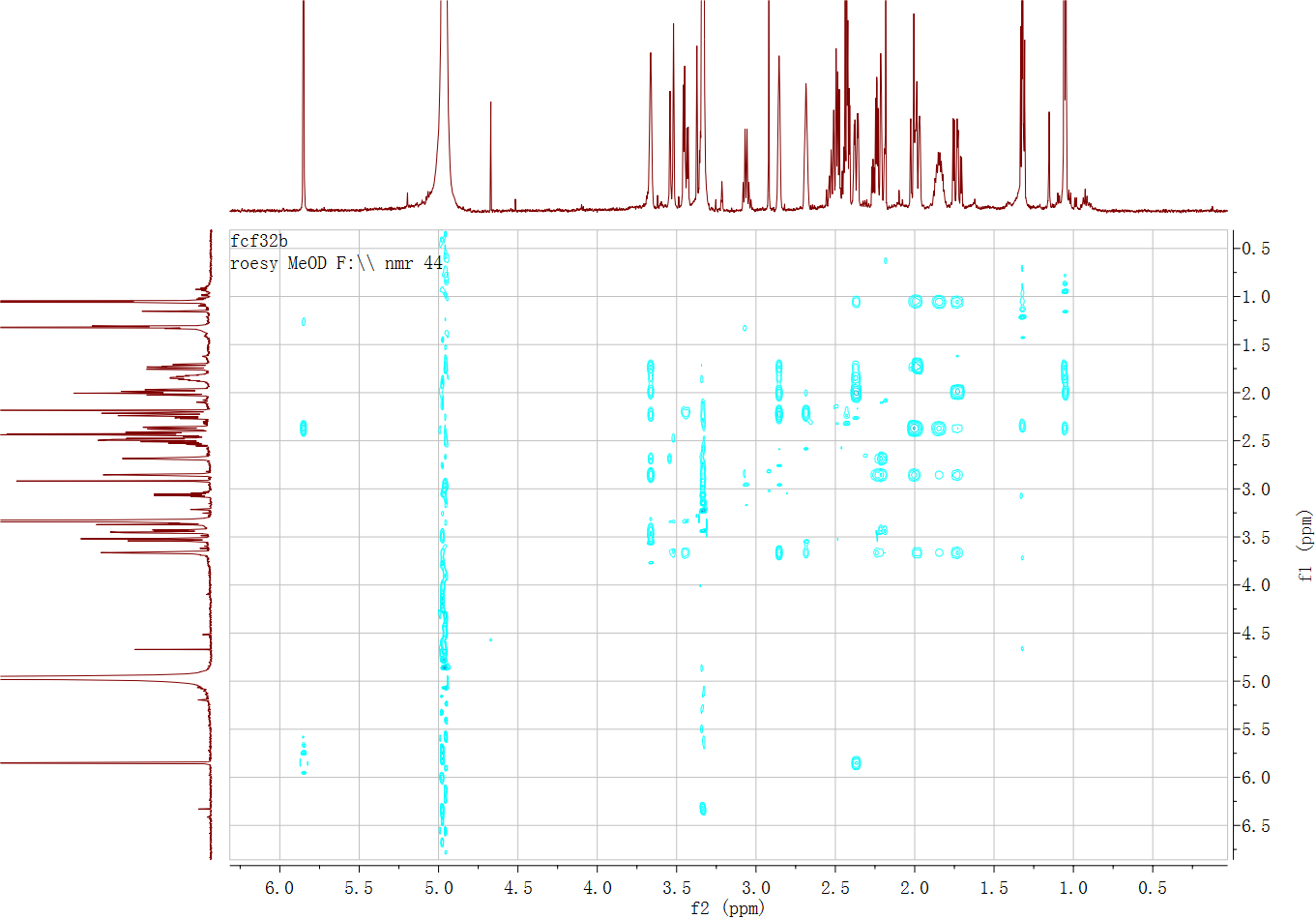

Supplement: Supplementary file 1 — Supplementary material 1 (DOCX 5689 kb) [file 13659_2016_111_MOESM1_ESM.docx]
